# Challenges and Future Perspectives of Immunotherapy in Pancreatic Cancer

**DOI:** 10.3390/cancers13164235

**Published:** 2021-08-23

**Authors:** Anna Maxi Wandmacher, Anne Letsch, Susanne Sebens

**Affiliations:** 1 Department of Internal Medicine II, University Medical Center Schleswig-Holstein, Campus Kiel, 24105 Kiel, Germany; annamaxi.wandmacher@uksh.de (A.M.W.); Anne.Letsch@uksh.de (A.L.); 2 Institute for Experimental Cancer Research, Kiel University and University Hospital Schleswig-Holstein, Campus Kiel, 24105 Kiel, Germany

**Keywords:** PDAC, immunosuppression, tumour microenvironment, cancer vaccine, checkpoint inhibition, translational research

## Abstract

**Simple Summary:**

Immunotherapeutic agents harness the patient’s immune system to fight cancer cells. Especially immune checkpoint inhibitors, a certain group of immunotherapeutic agents, have recently improved treatment options for many cancer types. Unfortunately, clinical trials testing of these agents in pancreatic cancer patients have not confirmed promising results from laboratory experiments. Several characteristics of pancreatic cancer biology, especially the profound tumour microenvironment that inhibits the successful identification and elimination of tumour cells by immune cells seems to be responsible for the lacking efficacy of immunotherapeutics in pancreatic cancer. We summarise recently published clinical trials investigating immunotherapeutic strategies in pancreatic cancer patients and available data on how these treatments influence pancreatic cancer biology. Moreover, we identify potential strategies to improve experimental and clinical studies in order to generate more conclusive data and improve patient outcomes in the future.

**Abstract:**

To date, extensive efforts to harness immunotherapeutic strategies for the treatment of pancreatic ductal adenocarcinoma (PDAC) have yielded disappointing results in clinical trials. These strategies mainly focused on cancer vaccines and immune checkpoint inhibitors alone or in combination with chemotherapeutic or targeted agents. However, the growing preclinical and clinical data sets from these efforts have established valuable insights into the immunological characteristics of PDAC biology. Most notable are the immunosuppressive role of the tumour microenvironment (TME) and PDAC’s characteristically poor immunogenicity resulting from tumour intrinsic features. Moreover, PDAC tumour heterogeneity has been increasingly well characterized and may additionally limit a “one-fits-all” immunotherapeutic strategy. In this review, we first outline mechanisms of immunosuppression and immune evasion in PDAC. Secondly, we summarize recently published data on preclinical and clinical efforts to establish immunotherapeutic strategies for the treatment of PDAC including diverse combinatorial treatment approaches aiming at overcoming this resistance towards immunotherapeutic strategies. Particularly, these combinatorial treatment approaches seek to concomitantly increase PDAC antigenicity, boost PDAC directed T-cell responses, and impair the immunosuppressive character of the TME in order to allow immunotherapeutic agents to unleash their full potential. Eventually, the thorough understanding of the currently available data on immunotherapeutic treatment strategies of PDAC will enable researchers and clinicians to develop improved treatment regimens and to design innovative clinical trials to overcome the pronounced immunosuppression of PDAC.

## 1. Introduction

Many cancer patients have benefited from novel immunotherapeutic approaches, most notably immune checkpoint inhibitors. This class of drugs harnesses the immune system for the fight against aberrant cancer cells by inhibiting co-inhibitory signals (immune checkpoints) allowing the undamped response of cytotoxic CD8+ T lymphocytes (CTL) to the cancer cells. Unfortunately, patients diagnosed with pancreatic ductal adenocarcinoma (PDAC) have to date not benefited from these and other immunotherapeutic innovations, suggesting the existence of comprehensive immune escape and immunosuppression mechanisms.

Regarding a projected increase of PDAC cases, PDAC is expected to be the second most common cause of cancer-related death by 2030 in the United States [1]. Still, therapeutic options are very limited, resulting in a 5-year overall survival with optimal care of 5–9% [2]. Due to the lack of specific symptoms and effective early detection measures, approximately 80% of patients are diagnosed with locally advanced or metastasised PDAC. Currently, the standard of care for this large group of patients is palliative chemotherapy with FOLFIRINOX (5-FU, Irinotecan, Leucovorin, and Oxaliplatin), or Gemcitabine/Nab-Paclitaxel. These therapies moderately improve overall survival to 11.1 months [3] and 8.5 months [4], respectively, in comparison to the previous standard of Gemcitabine monotherapy (6.8 months). However, not all patients are fit for these therapy regimens, and often adverse effects significantly reduce the quality of life. A minority of patients (approximately 20%) is diagnosed with a localised PDAC, being eligible for surgical removal of the primary tumour (if fit enough for the extensive surgical procedure), the only potentially curative treatment option [5]. However, even 80% of these patients who undergo surgical removal of the primary tumour followed by adjuvant chemotherapy with FOLFIRINOX experience relapse within the first five years after diagnosis, resulting in an overall median survival of 54.4 months [6]. Taken together, these data underscore the urgent need to improve therapeutic options for PDAC patients of all stages.

Immune surveillance essentially contributes to the elimination of altered (and potentially malignant) cells and thereby prevents cancer onset under physiological conditions [7]. Moreover, most solid tumours comprise a variety of immune cell populations, providing the rationale for immunotherapeutic strategies to eliminate or at least control tumour burden.

Accordingly, despite the lack of clinical efficacy to date, immunotherapeutic approaches represent the largest group of therapies currently under investigation for the treatment of PDAC patients [8]. Immunotherapeutic approaches under investigation are based on different mechanisms of action, such as activation of T-cell responses by monoclonal antibodies targeting immune checkpoints, vaccination, or adoptive immune-cell transfer. Since monotherapeutic strategies often fail, recent approaches aim at combining different immunomodulating agents, e.g., checkpoint inhibitors with agents that attract and activate antigen-presenting cells (chemokine agonists, cluster of differentiation 40 (CD40) agonists) or suppress factors favouring immune-suppressive cell signatures in the tumour microenvironment (TME) of PDAC (chemokine antagonists).

In this review, we outline the diverse mechanisms by which PDACs suppress and escape the activity of immune cells and summarise recently published data from preclinical and clinical trials exploring the therapeutic potential of immunotherapeutic strategies for the treatment of PDAC. As available preclinical and clinical data is rapidly mounting, we mainly focus on current data from the last three years with a special emphasis on the role of immune checkpoint inhibitors. Additionally, we outline some translational data obtained from the evaluation of patients treated with these therapies to identify recurrent patterns of immunologic responses that may be employed for improved combinatorial regimens. Finally, we highlight unresolved preclinical and clinical research questions arising from the latest developments of the field with a focus on standards for the translational evaluation of patient material, biomarker development, and the role of tumour heterogeneity for tailored treatment strategies.

## 2. Mechanisms of Immunosuppression and Immune Evasion in PDAC

Like other solid carcinomas, PDAC is characterised by high tumour heterogeneity, which refers to the different geno- and phenotypes of the PDAC cells, as well as to the tumour microenvironment (TME). The latter often comprises more than 80% of the total tumour mass and, in addition to a distinct desmoplastic response, a variety of stromal cells, such as carcinoma-associated fibroblasts (CAF), endothelial cells, and diverse immune cell populations are found [9]. Both the extent and phenotypes of stromal cells are subject to high dynamics, as they change during tumourigenesis and in turn critically influence it.

Importantly, a high number of CTL infiltrating the tumour tissue positively correlates with improved survival of PDAC patients, while a high number of regulatory FOXP3+ T-cells (Treg), functionally suppressing the activity of effector T-cells, correlates with poor outcomes [10]. Additionally, long-term survivors of PDAC exhibited a more diverse T-cell receptor (TCR) repertoire reflecting a T-cell response to multiple tumour antigens [11].

In order to successfully induce T-cell mediated elimination of cancer cells, several steps of the cancer-immunity cycle resulting in CTL activation are necessary. These have been comprehensively characterised by Chen et al. [7]. Tumour-specific (neo)antigens are required that allow the identification of aberrant tumour cells by immunosurveillance through resident antigen-presenting cells (APC). Typically, these antigens arise from the expression of mutated or aberrantly expressed genes and are released upon cancer cell death. To provoke proper CTL priming, antigens must be presented by dendritic cells via MHC I and II complexes. Then, T-cells interact with antigen-presenting cells (APC) resulting in their activation and proliferation if sufficient co-stimulatory signals are present. CTL traffic to the tumour site, infiltrate the tumour site and invade the tumour tissue, where they identify tumour cells. Finally, CTL eliminates tumour cells [12]. 

Even though PDAC tissues comprise T-cell populations to varying amounts, it is meanwhile well appreciated that PDAC evades T-cell mediated cytotoxicity and hampers the activation of adaptive immunity by different means. Thus, immune evasion starts right from the beginning of tumourigenesis as PDAC harbours a comparatively low tumour mutational burden compared to other cancers such as melanoma [13]. This results in a smaller number of neoantigens from aberrantly transcribed genes available as a starting point for an adaptive immune response. In terms of survival, a higher number of tumour-specific neoantigens correlates with a better prognosis in PDAC patients when accompanied by simultaneous CTL infiltration [11]. Additionally, PDAC downregulates the expression of HLA class I molecules and thus hides from identification by APC and T-cells [14]. An important immune evasion strategy in PDAC can be also seen in the pronounced desmoplastic stroma. CAF which are the most abundant inflammatory stroma cell population in PDAC release high amounts of extracellular matrix (ECM) proteins on the one hand [15] and a plethora of immunosuppressive factors on the other hand. This and a poor vascularisation of the tumour provide an efficient physical barrier characterised by increased interstitial fluid pressure [16] preventing the infiltration, expansion, and activity of T-cells in close proximity of PDAC cells [9]. This is further supported by the suppression of homing-receptors that allow T-cells to attach to the vessel wall and migrate into the tissue [17]. Finally, CTL that have eventually managed to enter the tumour stroma are faced by inhibitory signals released by a variety of other immunosuppressive cells, such as Treg, myeloid-derived suppressor cells (MDSCs) and M2-macrophages [18]. Altogether, these stromal cells dampen activity and expansion of T-cells, e.g., by secretion of inhibitory cytokines and chemokines (IL-4, IL-10, IL-13, IL-13, IL-17, transforming growth factor-beta (TGF-β)), remodelling of the ECM or by upregulation of immune checkpoints such as programmed cell death 1 ligand 1 (PD-L1) thereby preventing successful elimination of tumour cells [19]. In the latter context, it is important to note that in PDAC PD-L1 is predominantly expressed by stromal cells, e.g., macrophages [20,21]. Overall, PDAC is characterised by a variety of different immune evasion and immunosuppression strategies, and one of the major challenges is to identify the most prominent strategy in each individual patient in order to select the most effective treatment.

## 3. Strategies for Immunotherapy in PDAC

**Figure 1:**
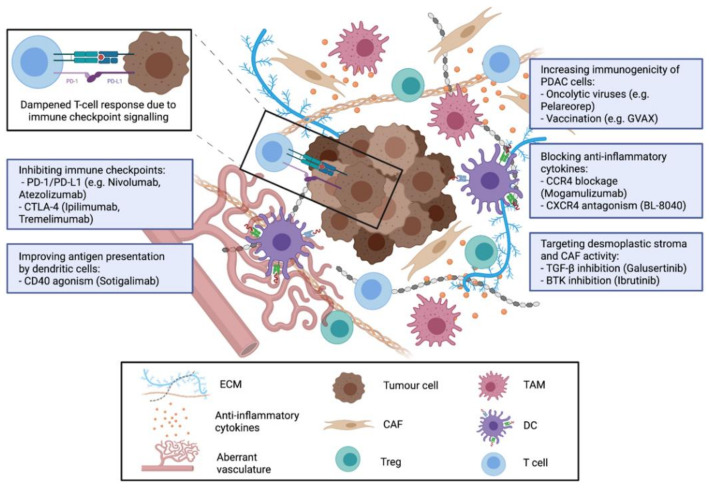
Therapeutic strategies to target immunosuppressive components of the TME in PDAC. Abbreviations: Extracellular matrix (ECM); cancer associated fibroblasts (CAF); regulatory T-cells (Treg); tumour associated macrophages (TAM); dendritic cells (DC). Figure created with BioRender.com.

The existing data support the rationale to develop immunotherapeutic regimens that increase the number and activity of tumour infiltrating lymphocytes (TIL) to elicit a potent anti-tumour T-cell response in PDAC patients. For this purpose, different strategies have been developed and explored for their efficacy in preclinical and clinical studies (Figure 1).

### 3.1. Cancer Vaccines for Treatment of PDAC

Applied as cancer therapy, cancer vaccines aim at the delivery of tumour-specific antigens to elicit a specific and strong T-cell mediated anti-tumour response whilst not inducing autoimmunity. The main obstacles towards an effective cancer vaccination strategy have been the choice of appropriate antigen(s), effective adjuvants, the mode of application [22], and combinatorial strategies. Accordingly, multiple peptides or whole cell-based tumour vaccines applied as single agents or in combination with conventional chemotherapy or additional treatment modalities have been explored for the treatment of PDAC. Antigens used for vaccination purposes have been, i.e., Mesothelin, mutated KRAS, Vascular Endothelial Growth Factor Receptors 1 and 2 (VEGFR1, VEGFR2), Kinesin-like Protein KIF20A and Wilms Tumour Protein 1 (WT1) [23,24,25].

GVAX is a cancer vaccine consisting of irradiated granulocyte-macrophage colony-stimulating factor (GM-CSF) secreting allogeneic pancreatic tumour cells. The induction of anti-tumour immunity by vaccination with irradiated GM-CSF expressing tumour cells had been explored preclinically in animal models for other cancers including melanoma and prostate cancer [26,27] and a pancreas-specific vaccine entered several clinical trials for evaluation of safety and efficacy. Translational data from a phase II clinical trial assessing GVAX alone or in combination with oral or intravenous cyclophosphamide administered perioperatively (first dose as neoadjuvant and additional doses as adjuvant treatment) in patients with resectable PDAC confirmed immunological effects of GVAX activating multiple immune cell populations in the tumour tissue of surgically resected specimens [28]. Tertiary lymphoid structures histologically resembling lymph nodes formed after GVAX treatment in the TME. On the one hand, GVAX treatment increased the number of Interferon-γ (INF-γ) secreting effector TIL, on the other hand also Treg increasingly infiltrated the TME upon treatment. Moreover, the expression of the immune checkpoint proteins programmed death protein 1 (PD-1) and its ligand PD-L1 increased in the tertiary lymphoid infiltrates but positively correlated with overall survival [28]. Final data from this phase II clinical trial reported a positive trend in mean overall survival (mOS) for patients treated with neoadjuvant GVAX alone (35.0 months compared to 24.8 months in historical controls) in comparison with patients who were additionally treated with cyclophosphamide (mOS 15.4 and 16.5 months) [29]. Moreover, the formation of tertiary lymphoid structures in the TME positively correlated with mOS suggesting a dominating role of the induced anti-tumour immunogenic activity over Treg invasion and increased immune checkpoint expression [29]. Of note, the trial was not powered for statistical comparison of treatment arms.

A second phase II clinical trial assessed GVAX in combination with low dose cyclophosphamide as a T-cell activity modulating agent alone or in combination with live attenuated mesothelin-expressing Listeria monocytogenes (CRS-207) for treatment of metastatic PDAC [30]. GVAX alone did not improve mOS. However, the addition of CRS-207 improved the mOS in this heavily pre-treated collective from 3.9 to 6.1 months warranting further clinical investigation [30]. A subsequent phase IIb study evaluating these results in a three-arm multicentre design with a larger cohort of patients (*n* = 213) did not confirm the initial positive results for a combination of GVAX and CRS-207 and this regimen did not outperform conventional chemotherapy [31]. Interestingly, translational research accompanying both trials identified two prognostic and predictive signatures of circulating immune-cells (CD8+CD45RO-CCR7-CD57+ and CD14+CD33+CD85j+) by multiplex flow cytometry and prospectively validated them. However, the functional relevance of these cell populations remains unclear [32].

Another vaccine approach for PDAC treatment is Algenpantucel-L, also known as the HAPa cancer vaccine, consisting of two allogeneic pancreatic cancer cell lines expressing the murine a(1,3)GT gene. Mechanistically, Algenpantucel-L aims at exploiting hyperacute rejection to murine proteins to induce a strong immune reaction towards PDAC peptides. This agent was clinically evaluated as part of a neoadjuvant treatment strategy in a phase III clinical trial (*n* = 303) adding it to a neoadjuvant standard of care chemotherapy and chemoradiation for the treatment of borderline resectable or locally advanced unresectable PDAC [33]. In contrast to promising data from a previous phase II clinical trial [34], the addition of Algenpantucel-L did not improve overall survival in this larger patient cohort. In terms of operability of the tumour, no significant differences were observed in patients receiving standard of care neoadjuvant treatment only or additional Algenpantucel-L (26% and 23%, respectively). Regarding the subgroup of patients who underwent surgical resection of the tumour, also no statistical differences in OS were observed (29.9 vs. 27.1 months). Unfortunately, no translational data was published to allow the assessment of immunological impacts of Algenpantucel-L treatment.

Researchers also evaluated tumour vaccination using OCV-C01, a peptide-based vaccine combining epitopes from KIF20A, VEGFR1, and VEGFR2, in PDAC patients. In a single-arm, open-label phase II trial with 30 participants the vaccine was evaluated in combination with Gemcitabine as an adjuvant therapy in patients with resected PDAC [23]. Translational data collected in this trial revealed a tendency towards longer survival in patients with specific CTL KIF20A peptide responses or detectable protein expression of KIF20A in surgically resected specimens. However, these results were not significant and due to the trial design, a potential benefit of the addition of OCV-C01 to Gemcitabine was difficult to assess.

Since KRAS is the most abundant mutated oncogene in PDAC found in approximately 90% of specimens, it represents an attractive target for tumour vaccination. Palmer et al. evaluated a KRAS-targeted peptide vaccine consisting of seven synthetic KRAS peptides representing the most common mutations found in PDAC (TG01/GM-CSF) in combination with Gemcitabine as an adjuvant treatment for stage I or II KRAS mutant PDAC in a phase I/II single-arm clinical trial [24]. The mean overall survival was 33.3 months compared to historical data sets evaluating Gemcitabine monotherapy (with mOS 17.1–26.5 months). Immune activation levels towards the provided antigens were evaluated by delayed hypersensitivity testing and in vitro T-cell assays from longitudinally collected blood samples. More than 90% of PDAC patients responded with either a positive immune response in delayed hypersensitivity testing or increased T-cell proliferation after stimulation with TG01/GM-CSF in vitro providing experimental evidence for the underlying therapeutic rationale.

Although several additional cancer vaccine strategies have been explored in clinical trials, none of them has significantly improved the survival and prognosis of PDAC patients [35,36,37,38]. One explanation might be that boosting of effector T-cells alone is not sufficient to overcome the existing immunosuppression in PDAC TME. Thus, more recent therapeutic approaches explore the efficacy of cancer vaccines combined with immune checkpoint inhibitors, which are outlined in the next section.

### 3.2. Immune Checkpoint Inhibitors for PDAC Treatment

Despite great hopes, checkpoint inhibitors targeting either PD-1, PD-L1 or cytotoxic T-lymphocyte-associated protein 4 (CTLA-4) applied as monotherapies have not yielded clinical improvements for PDAC patients in contrast to therapeutic successes in other cancers [39,40,41,42]. Thus, further efforts have focussed on the combination of checkpoint inhibitors with other therapeutic strategies, e.g., with conventional chemotherapeutic agents. Rationally, this combinatorial approach is based on a potential pro-immunogenic effect of chemotherapy exerted by increased release of tumour antigens and induction of an inflammatory milieu upon rapid cell death which is facilitated by a profound depletion of MDSCs and Treg from peripheral blood and tumour sites [43,44,45]. However, in the clinical setting, the combination of the CTLA-4 inhibitor Tremelimumab with Gemcitabine [46] or the combination of the PD-1 inhibitors Nivolumab or Pembrolizumab with Gemcitabine and Nab-Paclitaxel did not relevantly improve outcomes of PDAC patients in phase I clinical trials [47,48]. Wainberg et al. provided translational data on the effects of Nivolumab on immune cell populations in PDAC patients demonstrating that CD4+ and CD8+ T-cell populations in peripheral blood increasingly proliferated after exposure to Nivolumab as reflected by antigen Ki-67 (Ki-67) positivity. Additionally, T-cell proliferation positively correlated with clinical outcomes. However, a comparison of pre- and on-treatment biopsies by immunohistochemistry (IHC) revealed no changes in T-cell populations within the tumour hinting at a T-cell activating effect in the periphery but at a lack of T-cell infiltration into the tumour site [47].

To overcome the clinical inefficacy of checkpoint inhibitors for the treatment of PDAC, multiple combinatorial approaches to harness T-cell mediated responses have been clinically investigated. These include combinations of two checkpoint inhibitors simultaneously targeting the PD-1, PD-L1, and CTLA-4 axes (or triple therapy with stereotactic radiation), combined with small molecule inhibitors (i.e., TGF-β receptor 1 kinase or Bruton tyrosine kinase (BTK) inhibitors), combined with an oncolytic virus, combined with chemokine antagonists, with CD40 agonists or with cancer vaccines. This overview underscores the diversity of therapeutic approaches being used in the clinical trial landscape to overcome immunosuppression in PDAC patients, which is even broader considering ongoing or abstracted clinical trials (Table 1). These include combinations with IL-6 antagonist (NCT04258150), irreversible electroporation (NCT04212026), personalized tumour vaccines (NCT04161755, NCT03806309), a stimulator of interferon genes (STING) agonistic vaccine (NCT03010176), an inducible T-cell costimulator kinase inhibition (ICOS) targeted antibody (NCT03829501), kinase inhibitors (NCT04820179; NCT04820179), and CD73 antagonists (NCT03806309).

Two recently published clinical trials investigated checkpoint inhibition with Durvalumab (PD-L1 inhibitor) alone or in combination with Tremelimumab s(CTLA-4 inhibitor) for the treatment of recurrent or metastatic PDAC patients with [49] or without additional stereotactic body radiation [50]. The dual combination without additional radiation did not improve outcomes in a phase II study with 65 participants. Both experimental arms achieved an mOS of 3.6 and 3.1 months for Durvalumab alone or Durvalumab plus Tremelimumab, respectively [50]. In a phase I clinical trial with 58 participants, the addition of stereotactic body radiation to either Durvalumab monotherapy or combination therapy with Durvalumab and Tremelimumab resulted in a very modest clinical benefit for patients with metastatic PDAC treated with at least one line of previous systemic therapy [49]. As observed in other clinical trials investigating checkpoint inhibitors in PDAC patients, analysis of paired biopsies obtained at baseline and on treatment revealed a non-significant increase in CD3+ and CD8+ T-cells within the tumour. However, this did not correlate with outcome parameters and as it was only determined in five patients, the validity of this finding is quite limited.

TGF-β is a key player involved in creating and maintaining an immunosuppressive TME in PDAC, e.g., by inducing transdifferentiation and ECM production in CAF and inhibiting Granzyme B mediated T-cell cytotoxicity [51]. Based on these findings, combined TGF-β and PD-L1 inhibition was evaluated in a mouse model of immune excluded tumours and resulted in an increased invasion of CTL and tumour shrinkage [52]. These preclinical datasets supported the rationale for the combined inhibition of TGF-β and immune checkpoints for the treatment of PDAC. Melisi et al. investigated this therapeutic strategy combining the TGF-β receptor I kinase inhibitor Galunisertinib with PD-L1 inhibitor Durvalumab in 32 patients with metastatic or recurrent PDAC who had obtained up to two previous lines of therapy [53]. Combined treatment resulted in an overall response rate (ORR) of 3.1%, a disease control rate (DCR) of 25% and an mOS of 5.72 months. Importantly, PD-L1 expression did not correlate with the therapeutic response highlighting the complexity of biomarker identification for patient stratification.

Similarly, BTK signalling contributes to the generation of the immunosuppressive TME. Mechanistically, BTK signalling is involved in the polarisation of macrophages towards an M2-phenotype. Tumour-associated macrophages (TAM) often exhibit an immunosuppressive M2-subtype which is characterised by secretion of chemokines and cytokines dampening T-cell activity, facilitating polarisation of Treg, promoting angiogenesis, and inducing ECM production by CAF [54]. In a mouse model of PDAC, treatment with a BTK inhibitor increased the number of tumour-infiltrating CD8+ T-cells and resulted in tumour shrinkage. Antibody-mediated depletion of CD8+ T-cells reversed the anti-tumour effect of the BTK inhibitor and thus underscored the importance of CD8+ T cells for this therapeutic strategy [55]. Additionally, BTK signalling mediates desmoplasia of PDAC adding another rationale for BTK inhibition in PDAC treatment [56]. However, combined treatment with the BTK inhibitor Ibrutinib and Gemcitabine/ Nab-Paclitaxel did not improve outcomes of PDAC patients in comparison to chemotherapy alone in a phase III trial with 424 participants [57]. A smaller phase II trial (n=77) investigated the effect of the BTK inhibitor Acalabrutinib alone or in combination with the PD-1 inhibitor Pembrolizumab in patients with unresectable or metastatic PDAC who had at least obtained one previous line of systemic treatment [58]. Again, neither Acalabrutinib alone nor the combination yielded improvements in mOS (3.6 and 3.8 months, respectively) despite a significant reduction in granulocytic MDSCs observed in peripheral blood samples in both therapy arms.

Another approach to sensitise PDAC for checkpoint inhibition is the combination with oncolytic viruses. By inducing selective lysis of tumour cells, oncolytic viruses have the property to alter the characteristics of immunologically non-accessible tumours. Virus-induced cell lysis results in increased tumour antigen levels at the site of cell death and augments invasion and maturation of macrophages and dendritic cells that play a key role in recruiting and activating T-cells via chemokine secretion, mostly of INF-γ and TNF-α [59]. Based on this rationale, combinations of tumour vaccines with T-cell based therapies in xenograft models of melanoma and non-small cell lung cancer [28] or with immune checkpoint inhibitors in a mouse model of glioblastoma have been evaluated and resulted in improved survival of animals [60,61]. Additionally, treatment with the oncolytic reovirus Pelareorep resulted in upregulation of PD-L1 levels in PDAC patients treated in a phase II clinical trial underscoring the rationale for a combination with immune checkpoint inhibition [62]. To address this therapeutic approach, a phase Ib clinical trial (n=11) was performed evaluating the combination of Pelareorep, the PD-1 antagonist Pembrolizumab and chemotherapy with 5-FU, Gemcitabine, or Irinotecan [63]. Among patients with advanced or metastatic PDAC who had obtained one prior line of therapy, a DCR of 3/10 patients was observed resulting in an mOS of 3.1 months. Replicating virus was detected in most on-treatment biopsy samples. Moreover, treatment significantly increased levels of multiple chemokines (CXCL9, CXCL10, CXCL11) that are functionally involved in establishing an immunologically active state by attracting and activating leucocytes. This therapeutic regimen was further investigated in a phase II trial (NCT03723915). However, due to data from clinicaltrials.gov this trial did not meet interim analysis criteria for continuation [64].

As outlined above, Tregs are highly abundant in the TME and peripheral blood of PDAC patients even at the early stages of the disease and are key players in modulating the immune response in PDAC [65]. Mechanistically, they mainly suppress INF-γ production and thus hinder the activation of TIL. However, tumour-suppressing characteristics of Tregs have also been described [66]. PDAC (and other tumours) secrete high amounts of cytokines (CCL17; CCL22) which bind to the CC chemokine receptor 4 (CCR4) primarily expressed on Tregs. Thereby, PDAC cells attract higher numbers of Treg contributing to the immune suppressed TME [67]. Mogamulizumab is a monoclonal antibody blocking CCR4 and has been approved for the treatment of cutaneous T-cell lymphoma. Clinical evaluation has revealed a profound reduction of circulating and tumour associated Tregs in tumours upon treatment with Mogamulizumab in line with preclinical findings [68]. Based on these data, Mogamulizumab was hypothesised to enhance the effect of checkpoint inhibitors by facilitating effector T-cell activation in absence of inhibitory Treg. Initial evaluation of Mogamulizumab in combination with PD-1 inhibition by Nivolumab in advanced solid tumours (*n* = 90) in a phase I clinical trial revealed promising results for the PDAC cohort (15 patients with locally advanced or metastasised PDAC, at least one previous line of systemic treatment) [69]. In this clinical trial, a DCR of 40% and an mOS of 6.5 months were observed for combinatorial treatment of the PDAC cohort. While clinical efficacy did not correlate with PDL-1 or CCR4 expression levels on tumour cells, the number of TIL or the mutational burden, Mogamulizumab reduced the proportion of Treg and increased the proportion of CTL in on-treatment biopsies compared to baseline. However, these findings did not correlate with clinical outcomes. In contrast to these encouraging findings, data from a second phase I trial investigating Mogamulizumab in combination with the CTLA-4 inhibitor Tremelimumab or PD-L1 inhibitor Durvalumab yielded no convincing antitumour efficacy in various solid tumours including a PDAC expansion cohort (ORR 0%) (*n* = 24). However, an effective reduction of tumour-infiltrating Tregs in post-treatment biopsies compared to baseline could be monitored [70]. Overall, these data suggest that despite an effective reduction in Tregs and an increase in CTLs in PDAC tissue, this treatment did not result in pronounced anti-tumour effects and improved survival of PDAC patients. Furthermore, these results indicate that additional immunosuppressive mechanisms must be active in PDAC that compromise the efficacy of this therapeutic approach in this tumour entity.

Another recently explored combinatorial partner for checkpoint inhibition is BL-8040, a CXC chemokine receptor 4 (CXCR4) antagonist. CXCR4 is abundantly expressed on leucocytes and signalling via its ligand CXCL12 is functionally involved in chemotaxis and bone marrow homing of leucocytes. Pharmacological blockade of CXCR4 or abrogation of its main source CAFs by introducing a diphtheria toxin receptor-expressing Fap transgene leading to eradication of Fap expressing CAFs upon diphtheria toxin administration in a mouse model of PDAC altered the composition of the TME and increased T-cell invasion into the tumour. Furthermore, the combination with PD-1 inhibition resulted in tumour shrinkage in this mouse model [71]. Additionally, treatment of organotypic slice cultures from human PDAC specimens with combined BL-8040 and PD-1 inhibition caused a rapid CTL infiltration of the tumour and induction of tumour cell apoptosis [72]. Based on these preclinical findings, a phase II clinical was initiated to investigate the combination of BL-41080, Pembrolizumab and conventional chemotherapy (5-FU, Leucovorin, nanoliposomal Irinotecan) in patients with metastatic PDAC who had obtained previous systemic treatment [73]. Two cohorts were enrolled: Cohort 1 (*n* = 37) had received one or more lines of systemic treatment, cohort 2 (*n* = 22) one previous line with a Gemcitabine-based regimen. Both cohorts showed a DCR of 34.5% and 77%, respectively. In cohort 2 of less-heavily pre-treated patients, ORR was 32% and the mean duration of response was 7.8 months. Due to these data showing a promising modest improvement of outcomes, a clinical investigation is ongoing in a second clinical trial (NCT02907099). Translational findings from IHC profiling of paired pre- and post-treatment biopsies of 24 PDAC tissues showed increased infiltration of different T-cell populations, especially activated CD8+ T-cells and decreased numbers of granulocyte-like MDSCs.

Another approach to improve CTL mediated anti-tumour responses in PDAC represents the combination of the cancer vaccine GVAX (see above) and immune checkpoint inhibition. To this end, the safety and efficacy of GVAX in combination with the CTLA-4 inhibitor Ipilimumab were evaluated as a maintenance therapy in PDAC patients with stable disease or ongoing response after 8–12 doses of FOLFIRINOX in comparison to FOLFIRINOX continuation in a phase II clinical trial [74]. The mean overall survival after treatment with GVAX and Ipilimumab was 9.38 months compared to 14.7 months after continuation with FOLFIRINOX. These results indicated no clinical benefit from combined immunotherapy but clearly favoured FOLFIRINOX continuation for this specific patient cohort. Interestingly, analysis of peripheral blood comparing baseline to on-treatment samples revealed reduced numbers of naïve T-cells and increased numbers in T helper and T effector memory cells after GVAX and Ipilimumab treatment. Moreover, multiplex IHC of biopsy pairs obtained prior to and on treatment was performed and revealed a significant increase of CTL upon GVAX and Ipilimumab treatment. However, these alterations of the PDAC TME did apparently not translate into a clinical benefit, and it must be assumed that additional immunosuppressive mechanisms are still active in the TME dampening the activity of the different effector T-cells. In line with this assumption, treatment with GVAX and Ipilimumab resulted in an upregulation of the immune checkpoint T-cell Immunoglobulin and Mucin Domain-3 (TIM-3) in TIL highlighting complex compensatory mechanisms triggered by this therapeutic intervention [74]. Recently, results from a second clinical trial investigating the effect of GVAX in combination with immune checkpoint inhibition have been published. This phase II clinical trial investigated the safety and efficacy of GVAX/Cyclophosphamide/CRS-207 with or without additional Nivolumab (PD-1 inhibitor) in 93 patients with metastatic PDAC. In this patient cohort who had received one prior line of systemic therapy, the addition of Nivolumab did not significantly increase overall survival (+Nivolumab 6.1 months mOS versus -Nivolumab 5.9 months mOS) [75]. Multiplex IHC was performed on 22 biopsy pairs obtained prior to and on treatment identifying a significantly increased density of CD45+ leucocytes and an increased lymphoid to myeloid cell ratio in patients with long-term survival (>6 months). Moreover, short-term survivors (<6 months) were characterised by higher IL-6 levels [75]. Additionally, the T-cell repertoire of patients treated with Nivolumab was monitored over time demonstrating that long term survivors (> 6 months) exhibited significantly higher TCR clonalities after three cycles of treatment than patients with shorter survival [76].

Finally, another approach to potently activate immune responses against PDAC is combining immune checkpoint inhibition with CD40 agonists. CD40 is a receptor expressed primarily by DC, B-cells, and myeloid cells. Functionally, CD40 is activated by its ligand CD40L, which is mostly expressed on CD4+ helper T-cells and mediates the maturation of DC allowing them to activate CTL [77]. CD40/CD40L interaction is a proximal step in the activation of DC and B-cells resulting in a plethora of downstream effects. These include the increased expression of MHC-II and CD86 (a member of the immunoglobulin superfamily and a co-stimulator for T-cell activation) [78]. Functionally, this allows for increased antigen presentation accompanied by enhanced costimulatory signalling. In the KPC mouse model, CD40 agonism additionally results in macrophage activation also accompanied by increased MHC-II and CD86 expression mediating T-cell independent effects. Via increased secretion of CCL2 and INF-γ and elevated expression of matrix metalloproteinases, macrophages invade and reduce stromal matrix upon CD40 activation [78]. This reversal of tumour desmoplasia makes CD40 an attractive target for multiple therapeutic strategies in PDAC. Moreover, macrophages’ direct ability to lyse tumour cells upon CD40 agonist treatment has been demonstrated in the KPC mouse model [79]. Of note, the combination of a CD40 agonistic monoclonal antibody with PD-1 or CTLA-4 inhibition synergistically induced an anti-tumour immune response resulting in tumour regression in another mouse model of PDAC. This effect was largely reversed by abrogation of CD4+ or CD8+ T-cells underscoring the important role of effector T-cells in the anti-tumour effect [80].

Based on this rationale, multiple clinical trials have been conducted. First, Beatty et al. published a small phase I single-arm clinical trial (*n* = 21) investigating the CD40 agonist Selicrelumab (CP-870,893) in combination with Gemcitabine for the treatment of chemotherapy-naïve non-operable PDAC. Whilst tolerability was good, 4/21 patients experienced a partial response and 11/22 stable disease [79]. The resulting mOS of 7.4 months was better than historical controls with Gemcitabine monotherapy (5.7 months).

A recently published phase I clinical trial investigated the CD40 agonistic antibody Sotigalimab in combination with Gemcitabine/Nab-Paclitaxel with or without additional Nivolumab for the treatment of therapy-naïve metastatic PDAC (*n* = 30) [81]. The mOS across all cohorts was 20.1 months and dual and triple combinations showed a high ORR ranging between 67% and 83%. Longitudinal collection of blood samples for evaluation of immune cell populations with flow cytometry revealed an increase in circulating DCs and multiple T-cell subtypes including activated CD8+ T-cells upon treatment with Sotigalimab plus chemotherapy. The addition of Nivolumab did not significantly influence these effects. Based on these promising results in a small patient collective, the randomised phase II portion of this clinical trial was initiated (NCT03214250). Results presented at the American Society of Clinical Oncology 2021 annual meeting revealed an overall lower ORR of 33% and an mOS of 14.5 for Gemcitabine/Nab-Paclitaxel plus Sotigalimab than seen in the phase I trial and of 10.1 months for the triple combination with additional Nivolumab (ORR 31%) [82]. Interestingly, in this clinical trial Gemcitabine/Nab-Paclitaxel plus Nivolumab resulted in the best ORR of 50% with an mOS of 16.7 months. Biomarker evaluation is ongoing and may contribute to the understanding of these results.

## 4. Conclusions and Perspectives

Preclinical and clinical data on a plethora of immunotherapy concepts for PDAC treatment are rapidly mounting. The lack of clinical responses towards a multitude of immunotherapeutic approaches has again shed light on the specific characteristics of PDAC as well as the limitations and challenges of transferring innovative research findings into effective clinical treatment of PDAC patients.

While translation from preclinical models to clinical application is generally difficult, the lack of novel therapeutics entering the standard of care for PDAC might specifically highlight the limitations of available preclinical models of PDAC. As PDAC is characterised by a high proportion of dense tumour stroma and resulting cell–matrix and cell–cell interactions, meaningful models should allow studying these interactions. Moreover, patient cohorts subjected to novel treatment strategies have often been treated with at least one prior line of therapy including repeated drug exposure. Most tumour models do not recapitulate this characteristic which may significantly shape the response towards experimental therapeutic agents. Innovative in vitro models, e.g., patient-derived organoid co-culture models and ex vivo culture of tissue slices may help to overcome some of the limitations of 2D cell cultures as they enable tumour cell/immune cell interactions in the presence of experimental drugs and have been demonstrated to recapitulate drug effects observed in patients [83,84,85].

Moreover, increasingly ambitious translational research programmes have been developed that accompany clinical trials and improve the understanding of PDAC immunology even if clinical endpoints are not met. Ideally, these programmes will result in “reverse translation” by experimentally establishing concepts to overcome resistance towards immunotherapeutic strategies. However, in terms of translational methodology, a lack of established standards for patient material sampling and methods to evaluate immune responses becomes apparent. While peripheral blood samples are often collected longitudinally and blood cell populations are assessed by flow cytometry-based assays, only a subset of clinical trial protocols includes the longitudinal collection of tumour material for histological evaluation and functional analysis of TIL. This is understandable as tumour access is anatomically difficult in PDAC patients and repeated sampling is associated with an imminent risk of complications. However, a comprehensive comparison of pre- and on- or post-treatment biopsies may substantially improve the understanding of biological processes in general and in individual patients. Moreover, additional factors such as the gut and intratumoural microbiomes determine responses to immunotherapy [86,87] and have to date not been broadly addressed in translational studies. A consensus definition of standards for the translational investigation of patient samples obtained from clinical trials investigating immunotherapeutic strategies may help to generate more meaningful data and decipher the biological processes involved in therapeutic responses.

Summarising the available translational data sets from clinical trials presented in this review, a recurring pattern of immune response to diverse immunotherapeutic strategies becomes obvious. Multiple clinical trials testing the efficacy of immunotherapeutic strategies reported decreased Treg numbers or increased T-cell proliferation in peripheral blood or TME [24,70] or even increased numbers of TIL or CTL [69,75]. However, these immunogenic alterations rarely translated into meaningful clinical benefit and did not consistently correlate with improved clinical outcomes [49,74]. These findings highlight either the existence of additional mechanisms operating concomitantly in the patient and preventing effective tumour cell identification and eradication by TIL in PDAC or that the most effective immunosuppressive mechanism has not been targeted in the respective patient by the evaluated therapeutic approach. Thus, translational research efforts should aim at comprehensively dissecting the individual immunosuppressive as well as putative tumour reactive mechanistic arsenal in PDAC patients to improve recognition and cytolysis of tumour cells by TIL. In this context, Chen and Mellman have introduced the seminal concept of an immune set point [88]. Briefly, the immune set point is defined as the situation when stimulatory factors favouring an immune response against cancer cells overrule the counteracting inhibitory factors and tumour cells are successfully eradicated by TIL. The immune set point is determined by tumour intrinsic (mostly genetic and epigenetic characteristics), host-specific (alleles determining overall immune response towards immunogenic stimuli), and external factors (microbiome, environmental parameters). The lacking clinical efficacy of immune therapeutics in PDAC despite induced changes in immune cell populations indicates their inefficacy in terms of reaching the immune set point. To date, it remains unresolved which factors mainly hamper the successful immune response towards PDAC cells. However, a broadened understanding of respective factors is urgently needed to improve our concepts of PDAC biology in the context of immunotherapy and exploit it therapeutically in a personalised manner, most likely by combining multiple immunomodulating agents, adding immunomodulating agents to targeted agents (e.g., kinase inhibitors) or combining them with tumour stroma modifying agents.

Strikingly, none of the above-mentioned trials selected or stratified patients based on molecular or immunological criteria. This is surprising, as intra- and inter-tumour heterogeneity are well-defined characteristics of PDAC [89] and have resulted in extensive efforts to establish meaningful subtypes based on integrated analysis of genetic, histological, and clinical data [90,91,92,93]. Additionally, three different states of PDAC immunogenicity can be defined that partially overlap with the molecular subtypes [94]. (1) The immune escape phenotype is characterised by high numbers of Treg and M2-macrophages and is associated with poor prognosis. (2) The immune rich phenotype is characterized by high numbers of TIL similar to (3) the immune exhausted phenotype. Whilst TIL exert tumour cell cytotoxicity in the immune rich phenotype, counterregulatory mechanisms (e.g., upregulation of immune checkpoints) hamper effective anti-tumour immunity in the immune exhausted phenotype despite the presence of TIL. Based on gene expression data, PDACs can also be stratified according to TIL activity into low and high cytolytic tumours [95]. These characteristics are strongly associated with tumour subtypes based on genetic alterations underpinning a strong link between genomic alterations and immune phenotypes in PDAC [95]. Regarding this complex picture of PDAC biology, a “one-fits-all” therapeutic approach will probably not yield substantial therapeutic benefits and will likely need to be replaced by individualised therapeutic concepts based on meaningful biomarkers.

In summary, the extensive efforts to investigate immunotherapeutic strategies for the treatment of PDAC have resulted in important insights into PDAC immunology and the plethora of ongoing clinical trials will add to this repository (Table 1). However, the mostly disappointing clinical results have also highlighted several pitfalls and future tasks for researchers and clinicians. These are namely (i) the careful choice and development of preclinical models mimicking best PDAC biology and immunity to ensure translatability of preclinical findings into meaningful clinical advances, (ii) the need to define standards for translational evaluation of clinical trials investigating immunotherapeutic treatments in PDAC to allow more comprehensive analyses of patient materials, (iii) the need to improve our understanding of the cancer immune cycle and the immune set point in PDAC (patients) to translationally overcome barriers towards efficient CTL responses, and (iv) the identification of biomarkers allowing the stratification of patients according to PDAC (immune) subtypes and study designs that acknowledge PDAC tumour (and host) heterogeneity. Consideration of these aspects will hopefully improve immunotherapeutic treatment and survival of PDAC patients as already achieved in other cancer entities.

## Figures and Tables

**Table 1:** Selected ongoing clinical trials investigating novel immunotherapeutic treatment strategies for PDAC. Abbreviations: (CY) Cyclophosphamide; (5-FU) 5-Fluorouracil; (Gem) Gemcitabine; (ICOS) Inducible T-cell costimulator kinase inhibition; (IRE) Irreversible electroporation; (MIS-MWA) Minimally invasive surgical microwave ablation; (Nab-Pacli) Nab-Paclitaxel; (RT) Radiotherapy; (STING) Stimulator of interferon genes.

Mechanism	Target	Compound	Combination	Phase	Identifier
Chemokine antagonism	IL-2	XB2001	Irinotecan, 5-FU, Leucovorin	I/II	NCT04825288
	IL-6	Siltuximab	Spartalizumab	I/II	NCT04191421
	CSF1	ARRY-382	Pembrolizumab	I/II	NCT02880371
	IL-1β	Canakinumab	Nab-Pacli, Gem, Spartalizumab	I	NCT04581343
	CD73	CPI-006	Ciforadenant or Nivolumab	I	NCT03454451
Immunostimulatory agonism	CD40	CDX-1140	CDX-301	II	NCT04536077
	CD40	ABBV-927	Budigalimab, mFOLFORINOX	II	NCT04807972
	IL-12	M9241	M7824 or M7824, RT	I/II	NCT03849469
	STING	MK-1454	Pembrolizumab	I	NCT03010176
Vaccination	Individual TA	RO7198457	Atezolizumab, mFOLFIRINOX	I	NCT04161755
	Multiple Tas	GVAX	CY, Pembrolizumab, IMC-CS4	Early I	NCT03153410
	Multiple TAs	GVAX	CY or CY, Nivolumab or CY, Nivolumab, Urelumab	I/II	NCT02451982
	Multiple TAs	GVAX	CY, Nivolumab, RT	II	NCT03161379
	Multiple TAs	GVAX	CY, Pembrolizumab, RT	II	NCT02648282
	KRAS	KRAS peptide vaccine	Nivolumab, Ipilimumab	I	NCT04117087
	Individual TA	Personalized neoantigen DNA vaccine		I	NCT03122106
	Multiple TAs	PANC 10.05 pcDNA-1/GM-Neo and PANC 6.03 pcDNA-1 neo vaccine	Alone or CY i.v. or CY orally	II	NCT01088789
	Ten TAs	OSE2101	Alone or Nivolumab	II	NCT03806309
Oncolytic virus	Cancer Cell	OH2 Herpes simplex virus		I/II	NCT04637698
	Cancer Cell	Talimogene laherparepvec		I	NCT03086642
	Cancer Cell	TBI-1401(HF10)	Nab-Pacli, Gem, S-1	I	NCT03252808
Immune checkpoint inhibition	PD-1	Durvalumab	Plerixafor	II	NCT04177810
	PD-1	Dostarlimab	Niraparib, RT	II	NCT04409002
	PD-1	Camrelizumab	Gem, MIS-MWA	II	NCT04156087
	PD-1	Cemiplimab	Nab-Pacli, Gem	II	NCT04498689
	PD-1	Pembrolizumab	RT	I/II	NCT02305186
	PD-1	Pembrolizumab	Lenvatinib	II	NCT04887805
	PD-1	Pembrolizumab	Epacadostat, CRS-207 or Epacadostat, CRS-207, GVAX, CY	II	NCT03006302
	PD-1	Pembrolizumab	Azacitidine	II	NCT03264404
	PD-1	Nivolumab	Losartan, FOLFIRINOX, RT	II	NCT03563248
	PD-1	Nivolumab	IRE	II	NCT04212026
	PD-1	Nivolumab	None or IRE or IRE, TLR-9	I	NCT04612530
	PD-1	Nivolumab	Nab-Pacli, Gem, Paricalcitol	I	NCT03519308
	PD-1	Nivolumab	Gem, S1	II	NCT04377048
	PD-1	Nivolumab	RT or RT, Ipilimumab	II	NCT02866383
	PD-1+CTLA-4	Nivolumab, Ipilimumab	RT	II	NCT04361162
	PD-1+CTLA-4	Nivolumab, Ipilimumab	RT, Tocilizumab	II	NCT04258150
	PD-1+CTLA-4	Nivolumab, Ipilimumab	RT, Nab-Pacli, Gem	I/II	NCT04247165
	PD-1+CTLA-4	Nivolumab, Ipilimumab	RT	II	NCT03104439
	PD-1+CTLA-4	Nivolumab, Ipilimumab	CRS-207 or CRS-207, GVAX, CY	II	NCT03190265
	PD-1+CTLA-4	Nivolumab, Ipilimumab	Maraviroc	I	NCT04721301
	PD-1+CTLA-4	Durvalumab, Tremelimumab	RT, Gem	II	NCT03572400
	PD-L1	Atezolizumab	Nab-Pacli, Gem, Selicrelumab or Nab-Pacli, Gem, Bevacizumab or Nab-Pacli, Gem, AB928 or Nab-Pacli, Gem, Tiragolumab or Cobimetinib or PEGPH20 or BL-8040 or RO6874281 or Nab-Pacli, Gem, Tocilizumab	I/II	NCT03193190
	PD-L1	Atezolizumab	Cabozantinib	II	NCT04820179
	PD-L1+TGF-β	SHR-1701	Nab-Pacli, Gem	Ib/II	NCT04624217
	ICOS	KY1044	Alone or Atezolizumab	I/II	NCT03829501

## Abbreviations

APC	Antigen presenting cell
BTK	Bruton Tyrosine Kinase
CAF	Carcinoma associated fibroblasts
CD40	Cluster of Differentiation 40
CTL	Cytotoxic T lymphocytes
CTLA-4	Cytotoxic T-Lymphocyte-Associated Protein 4
DCR	Disease control rate
ECM	Extracellular matrix
FOLFIRINOX	5-FU, Irinotecan, Leucovorin, Oxaliplatin
5-FU	5-Fluorouracil
GM-CSF	Granulocyte-Macrophage Colony Stimulating Factor
ICOS	Inducible T-cell costimulator kinase inhibition
IHC	immunohistochemistry
INF-γ	Interferon-gamma
KIF20A	Kinesine-like Protein KIF 20A
MDSC	Myeloid derived suppressor cells
mOS	Mean overall survival
ORR	Overall response rate
PD-1	Programmed cell death protein 1
PDAC	Pancreatic ductal adenocarcinoma
PD-L1	Programmed cell death 1 ligand 1
STING	Stimulator of interferon genes
TAM	Tumour-associated macrophages
TCR	T-cell receptor
TGF-β	Transforming Growth Factor-beta
TIL	Tumour infiltrating lymphocytes
TME	Tumour microenvironment
Treg	regulatory T-cells
VEGFR	Vascular Endothelial Growth Factor Receptor
